# Development and Validation of a Lifestyle-Based 10-Year Risk Prediction Model of Colorectal Cancer for Early Stratification: Evidence from a Longitudinal Screening Cohort in China

**DOI:** 10.3390/nu17111898

**Published:** 2025-05-31

**Authors:** Jialu Pu, Baoliang Zhou, Ye Yao, Zhenyu Wu, Yu Wen, Rong Xu, Huilin Xu

**Affiliations:** 1 Department of Biostatistics, School of Public Health, Fudan University, Shanghai 200032, China; 2 School of Life Science and Technology, ShanghaiTech University, Shanghai 201210, China; 3 School of Food Science and Engineering, Yangzhou University, Yangzhou 225127, China; 4 Shanghai Minhang Center for Disease Control and Prevention, Shanghai 200125, China

**Keywords:** colorectal cancer, risk prediction, lifestyle factors, dietary patterns, risk stratification

## Abstract

**Background:** Colorectal cancer (CRC) remains one of the leading causes of cancer-related mortality worldwide, with growing evidence linking risk to lifestyle and dietary factors. However, nutrition-related exposures have rarely been integrated into existing CRC risk prediction models. This study aimed to develop and validate a lifestyle-based 10-year CRC risk prediction model using longitudinal data from a large-scale population-based screening cohort to facilitate early risk stratification and personalized screening strategies. **Methods**: Data were obtained from 21,358 individuals participating in a CRC screening program in Shanghai, China, with over 10 years of active follow-up until 30 June 2021. Of these participants, 16,782 aged ≥40 years were used for model development, and 4576 for external validation. Predictors were selected using random survival forest (RSF) and elastic net methods, and the final model was developed using Cox regression. Machine learning approaches (RSF and XGBoost) were additionally applied for performance comparison. Model performance was evaluated through discrimination, calibration, and decision curve analysis (DCA). **Results**: The final model incorporated twelve predictors: age, gender, family history of CRC, diabetes, fecal immunochemical test (FIT) results, and seven lifestyle-related factors (smoking, alcohol use, body shape, red meat intake, fried food intake, pickled food intake, and fruit and vegetable intake). Compared to the baseline demographic-only model (C-index = 0.622; 95% CI: 0.589–0.657), the addition of FIT improved discrimination, and further inclusion of dietary and lifestyle variables significantly enhanced the model’s predictive accuracy (C-index = 0.718; 95% CI: 0.682–0.762; ΔC-index = 0.096, *p* = 0.003). **Conclusions**: Incorporating dietary and lifestyle variables improved CRC risk stratification. These findings highlight the value of dietary factors in informing personalized screening decisions and providing an evidence-based foundation for targeted preventive interventions.

## 1. Introduction

Colorectal cancer (CRC) ranks as the third most common malignancy and the second leading cause of cancer-related mortality worldwide, with over 1.9 million new diagnoses and approximately 935,000 deaths attributed to it annually [1]. China currently bears the highest burden of CRC incidence and mortality worldwide. Concerningly, this burden is projected to increase, driven by the rising incidence among younger populations and continued aging of the overall population [2,3]. Population-based screening has been shown to be effective in reducing both the incidence and mortality of CRC, primarily through the early detection and removal of premalignant adenomas, as well as the early diagnosis and treatment of cancers at more treatable stages [4,5,6]. However, despite being the gold standard for CRC detection, colonoscopy is invasive, costly, and time-consuming, presenting substantial challenges for its large-scale implementation. A risk-tailored approach has been proposed to reduce medical costs by targeting colonoscopy for high-risk individuals identified through either a positive fecal immunochemical test (FIT) result or an elevated integral risk score, which has the potential to improve cost-effectiveness, particularly in resource-limited settings [7,8]. Moreover, a better understanding of cancer risk may enhance informed consent and increase screening participation rates.

A variety of factors are associated with the incidence of CRC, including inherent factors like age, gender, genetics, and modifiable lifestyle behaviors, such as smoking and alcohol consumption [1,9,10,11]. Recent studies have increasingly highlighted the link between dietary intake and CRC risk. High consumption of red and processed meats has been consistently associated with an elevated risk of CRC, with a dose–response relationship observed for each additional 30 g/day of intake [12,13]. Conversely, diets rich in calcium, dairy products (e.g., milk and yogurt), whole grains, and fiber have demonstrated protective effects, reducing CRC risk by up to 17% for calcium and 14% for dairy products [14,15]. Integrating these factors into personalized CRC risk scores could help identify high-risk individuals and encourage the adoption of healthier lifestyle changes.

Many colorectal cancer (CRC) risk prediction models have been developed over the years, among which the Asia-Pacific Colorectal Screening (APCS) score is one of the most widely validated in Asian populations [16,17,18]. This model, incorporating age, gender, smoking status, and family history of CRC, provides a simple yet practical approach for risk stratification. However, its discriminatory ability remains limited, with an AUC of only 0.64. Additionally, many recently developed models rely on binary outcomes derived from cross-sectional or short-term follow-up data, which do not account for the long latency period of CRC development and may introduce biases in prediction accuracy [11,19,20]. Although some studies have analyzed long-term survival distributions, they often fail to incorporate time-to-event machine learning (ML) models, despite their potential advantages [21,22,23]. Unlike traditional approaches such as Cox proportional hazards (CPH) regression, machine learning models can flexibly capture complex nonlinear relationships and interactions among variables without requiring predefined assumptions, offering improved adaptability and predictive performance [24,25,26].

We attempted to develop a prediction model that incorporates dietary habits alongside established factors, using data from a prospective cohort with a median follow-up of 11 years. Both traditional CPH and time-to-event ML methods were applied to compare the performance and obtain reliable estimates. Finally, we displayed the models’ risk stratification capabilities and net benefit across the entire population to access their generalizability and potential clinical utility in guiding screening referrals. To our knowledge, few studies have integrated long-term follow-up dietary data with time-to-event machine learning models for CRC risk prediction, particularly in Asian populations. By addressing this gap, our study offers novel insights into personalized screening strategies and highlights the potential for more efficient allocation of colonoscopy resources.

## 2. Materials and Methods

We used the APCS model as the base model. To evaluate the incremental predictive value of additional risk factors, we first incorporated FIT results into the model. Subsequently, a comprehensive model was developed by integrating APCS variables, FIT outcomes, and dietary factors. All models were trained to predict the 10-year risk of colorectal cancer among individuals within the recommended screening age range. Model performance was evaluated using standard discrimination and calibration metrics.

### 2.1. Data Source

The dataset was obtained from a subset of participants in the Shanghai Community-based Colorectal Cancer Screening Program (SHcsp), a large-scale, government-funded initiative designed to promote the early detection of CRC among urban residents in China [27,28,29]. We used information from Minghang Area, a representative district of Shanghai. Between 2008 and 2012, residents aged 50–74 were invited as the target population to complete a two-sample qualitative FIT, alongside a structured risk assessment (RA) via face-to-face interviews at enrollment. The RA included demographic, lifestyle, and medical history information collected by trained investigators. Dietary intake was assessed using a structured food frequency questionnaire (FFQ), where participants reported the frequency and approximate quantity of consumption over the past 7 days. Visual aids, such as standardized bowls and gram reference charts, were provided to support estimation. Anthropometric measurements, including height, weight, waist circumference, and hip circumference, were measured in light clothing by trained staff using standardized protocols in accordance with WHO guidelines [30]. Diabetes status was obtained from the local electronic health record (EHR) system and defined as a documented diagnosis by a licensed physician, in accordance with national clinical guidelines.

Since the program was delivered as part of the public health services, individuals outside the target age range were also permitted to participate on a voluntary basis. Throughout the follow-up period, community physicians actively monitored participants by routinely collecting examination results from designated hospitals and maintaining regular participant contact. CRC diagnoses were continuously identified through linkage to the Shanghai Cancer Registry. Follow-up for cancer outcomes was last updated on 30 June 2021. The study received ethical approval from the Shanghai CDC (EC-P-2012-002), and written informed consent was obtained from all participants.

### 2.2. Study Population

**Figure 1:**
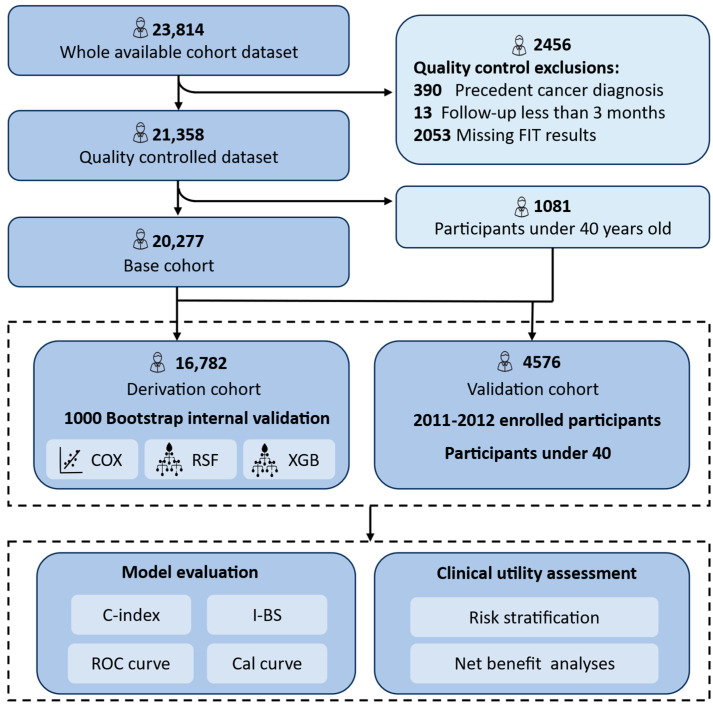
Flow diagram of the main steps in this study. The model was developed and internally validated using participants aged ≥40 enrolled during 2008–2010. External validation included those enrolled in 2010–2012 and participants <40 from the earlier cohort to increase sample size. All participants were followed up for over 10 years. Cox proportional hazards model, Cox; random survival forest, RSF; Extreme Gradient Boosting Accelerated Failure Time Model, XGB; Concordance Index, C-index; integrated Brier score, I-BS; Receiver Operating Characteristics, ROC; Calibration curve, Cal curve.

Of the initial 23,814 registered volunteers (aged 23–79 years), 21,634 participants remained after excluding those with missing FIT results, a prior cancer diagnosis, or follow-up durations shorter than three months. For model development and validation, we focused on 20,277 participants aged over 40 years—aligned with CRC screening guidelines by the Chinese CDC and international recommendations [31]. A training and internal validation cohort (*n* = 16,872) comprised individuals enrolled from 2008 to 2010. External validation was conducted on 3405 individuals recruited from 2011 to 2012. Additionally, 1081 participants under 40 years of age were included to ensure conservative model validation and improve robustness given the lower CRC incidence in younger populations (Figure 1).

### 2.3. Outcomes

The primary outcome was incident CRC, identified via linkage to the Shanghai Tumor Registration System. Cases of CRC were identified according to the International Classification of Diseases, 10th Revision (ICD-10) codes C18.0, C18.2–C18.9, C19.9, C20.9, while appendix cancer (C18.1) was excluded. For individuals with multiple primary CRC diagnosis, only the first occurrence was considered. Follow-up commenced at enrollment and continued until the first occurrence of CRC diagnosis, death, emigration, or 30 June 2021.

### 2.4. Sample Size Calculation

To reduce the risk of overfitting and potential biases arising from an insufficient sample size during variable selection and model training, we performed a preliminary sample size calculation. Assuming 30 predictor parameters, an annual incidence rate of 23.9 per 100,000 person years, a conservative Cox–Snell R^2^ of 0.03, and a 10-year prediction horizon with a mean follow-up time of 10.98 years, we estimated that the minimum sample size required was 10,822, corresponding to 118,825.6 person-time of follow-up (i.e., 10,822 × 10.98), with 29 outcome events and therefore an EPP (events per candidate predictor parameter) = 0.97. According to the guidelines by Riley et al., this value ensured a sufficient precision, a small shrinkage of predictor effects and optimism in apparent model fit [32,33,34].

### 2.5. Missing Data and Feature Processing

To mitigate multicollinearity and reduce model complexity, standardized BMI, waist circumference, and hip circumference were subjected to principal component analysis (PCA). The first principal component, which accounted for 93.6% of the total variance, was used to construct a composite variable referred to as “Body shape”. The calculation formula used in our study was as follows:

(1) Body shape=0.574×BMI+0.579×Waist+0.578×Hip

All input variables were standardized (z-score transformed) prior to PCA. These loadings were derived from the training dataset (*n* = 16,872) and were consistently applied throughout model development and validation.

Features with more than 30% missing data were excluded from subsequent analysis in the full dataset. For variables with less than 30% missingness, a total of 697 missing values were assumed to be missing at random and imputed using multiple imputation by chained equations (MICE) [35]. Continuous and nominal categorical variables were imputed using random forest, while ordinal categories were handled with proportional odds models. The imputation model included all preprocessed candidate predictors, the event indicator, and the Nelson–Aalen estimator of the cumulative hazard. Five imputed datasets were generated and subsequently used for model fitting and evaluation [35]. Details of missingness and imputation quality are provided in Supplementary Table S1 and Figure S1.

### 2.6. Feature Selection

Feature selection was performed using a dual-method approach, integrating regression-based elastic net regularization and tree-based random survival forest (RSF) [36]. The elastic net regularization, a hybrid method combining LASSO (L1 regularization) and ridge regression (L2 regularization), was implemented with optimal tuning parameters (λ and α) determined through 10-fold cross-validation, minimizing the Cox partial likelihood loss function [37]. Predictors with coefficient estimates shrinking to zero under these parameters were removed.

In the RSF approach, an ensemble extension of random forest, survival trees were built using bootstrapped samples, with survival functions estimated by averaging terminal node statistics [38]. Variable importance was assessed via permutation-based importance scores, with optimal number of features determined through 10-fold cross-validation targeting minimal error rates. To ensure robustness and consistency, feature selection was conducted independently on each imputed dataset, with results subsequently pooled using Rubin’s rules to generate unified feature importance metrics [39,40]. The final feature set consisted of predictors selected by both methods, ensuring the development of parsimonious models that incorporate only clinically relevant and readily accessible predictors.

### 2.7. Model Development

We use APCS as the base model. To evaluate the incremental predictive value of additional variables, FIT and lifestyle factors were sequentially incorporated to construct the FIT-enhanced and full models, respectively. All of the three models were constructed using CPH regression to estimate CRC risk. The proportional hazards (PHs) assumption was tested using Schoenfeld residuals for each covariate and globally, following model estimation. Additionally, the full model was further explored using RSF and XGBoost to explore potential nonlinear relationships and interactions among predictors [41,42]. All models were trained and internally validated using 1000 bootstrap samples on the training dataset. For the machine learning models, we employed a grid search with stratified 10-fold cross-validation to optimize their parameters. The best-performing parameters were then applied to the external dataset for model validation and evaluation. Detailed information about the hyperparameter search space is provided in Supplementary Table S2.

In the CoxPH model, the risk score is directly obtained from the linear predictor (lp), a weighted sum of covariates and their corresponding regression coefficients. RSF computes risk score by averaging the predicted cumulative hazard function (CHF) across all survival trees. In XGBoost, survival risk is estimated using a gradient-boosted tree, and the risk score is calculated as the sum of predictions from individual trees.

To derive the 5-year and 10-year risk estimates, time-dependent risk scores were transformed into cumulative risk probabilities. Specifically, for the CoxPH model, the survival probability at time *t* was estimated using the baseline survival function S_0_ adjusted by the exponentiated linear predictor. For RSF and XGBoost, the Nelson–Aalen estimator was used to estimate the cumulative hazard function (CHF), which was then converted to survival probabilities. All risk predictions were performed using the mlr3 and mlr3proba frameworks [43]. The final risk scores were standardized to ensure a consistent and comparable prediction across different models.

### 2.8. Model Evaluation

Model discrimination was assessed using Harrell’s C-index, calculated at 10 years, which quantifies the model’s ability to correctly rank the relative risk of two randomly selected individuals [44,45]. To account for model optimism due to overfitting, the C-index for each model was computed for every bootstrap sample, and the mean value was taken as an estimate of optimism. Optimism-corrected C-index was then calculated as C_apparent_ − Optimism [46,47]. The apparent C-index, bootstrap aggregated C-index with its 95% CI, optimism-corrected C-index were reported. Additionally, time-dependent ROC curves for each model at 5-year and 10-year time points were generated in the validation cohort to visualize their discriminative power. The analyses were performed in R with the package rms (version 5.1-3.1).

Model calibration was evaluated with the integrated Brier score (IBS) [48,49]. The Brier score measures the average squared distances between the observed survival outcomes and predicted survival probabilities. A lower IBS indicates better calibration. The metrics was computed using the mlr3proba package in R, which accommodates right-censored data [43]. Each model was internally validated using 1000 bootstrap samples, and the optimism in the calibration was adjusted similarly to the C-index. Calibration curves were generated for each model at 5-year and 10-year time points in the validation cohort.

To compare the clinical utility of the models, we assessed their performance across different risk strata and thresholds. We assumed a simplified scenario that a single colonoscopy at the cohort entry would detect all existing CRC cases. Net benefit (*NB*) was manually calculated in R using the standard formula [50]:

*NB* = (*TP* ÷ *N*) − (*FP* ÷ *N*) × (*Pt* ÷ [1 − Pt]) (2)

where *TP* = true positives, *FP* = false positives, *N* = total cohort size, and *Pt* = risk threshold (e.g., *Pt* = 1% implies willingness to perform 100 colonoscopies per detected case). Analyses were evaluated across a range of relevant risk thresholds.

## 3. Results

### 3.1. Baseline Characteristics

Table 1 summarizes the baseline characteristics of the study participants. The cohort was stratified by the decade of enrollment for model derivation and validation. Over the entire follow-up period (median: 11.41 years for the derivation cohort and 10 years for the validation cohort), a total of 285 CRC cases were observed. Compared to the development cohort, the validation cohort had a higher level of education, fewer FIT-positive cases, and a younger age profile due to later enrollment and the inclusion of 1180 individuals under age 40. The observed heterogeneity between cohorts helped assess model robustness under real-world conditions.

### 3.2. Selected Predictors

Supplementary Table S4 shows the distribution of Cox regression coefficients of all predictor variables based on the 10-fold cross-validated elastic net regularization. Variables whose coefficients were not shrunk to 0 were selected. For RSF, the best parameters were identified through grid search, with the model’s error rate stabilizing at approximately 0.35 after around 200 trees, indicating robust performance (Supplementary Figure S2). Top 12 variables were selected. The number of variables determined was the one that minimized the loss function plus one standard deviation in 10-fold cross validation.

The two methods produced consistent results with slight differences in variable ranking. Most predictors were significantly associated with the outcome. Variables with low prevalence, such as gastrointestinal symptoms, contributed minimally and were excluded from the final model. Ultimately, twelve variables were selected for the full model, including age, gender, smoking, family history, alcohol use, body-shape (first principal component), dietary factors, diabetes, and FIT. Table 2 presents the hazard ratios and 95% confidence intervals from the final multivariable Cox model. The proportional hazards (PHs) assumption was evaluated using Schoenfeld residuals, and detailed test results are provided in Supplementary Table S3.

### 3.3. Model Performance

With a C-index of 0.727 (bootstrap 95% CI: 0.682–0.762; Table 3) and a 10-year AUC of 0.811, the full model exhibits the highest discrimination performance among the three models. Compared with the base model (C-index = 0.626; 95% CI from bootstrap: 0.589–0.657), consistent with the original study), the addition of FIT increased the C-index by 0.073 (0.065 in the validation set), while the full model improved the C-index by 0.101 (0.096 in the validation set). These findings indicate a statistically significant enhancement in discrimination.

**Figure 2:**
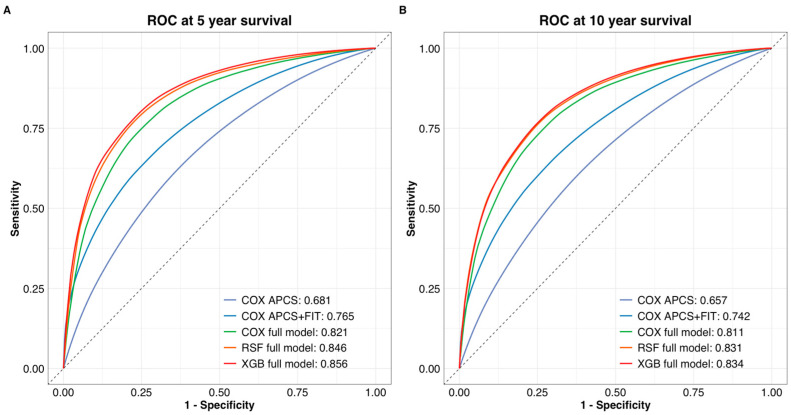
Time-dependent ROC curves for (**A**) 5-year and (**B**) 10-year colorectal cancer survival prediction. The Cox full model showed improved discrimination over the base and FIT-enhanced models. XGBoost achieved the highest AUC at both time points, followed by RSF. ROC curves were generated using the validation cohort.

The ranking of model performance based on time-dependent AUC was consistent with the C-index results. At both 5-year and 10-year time points, the full Cox model outperformed the base and FIT-enhanced models (AUC = 0.821 and 0.811, respectively). Machine learning models further improved discrimination, with XGBoost achieving the highest AUCs (0.856 at 5 years; 0.834 at 10 years), followed closely by RSF (Figure 2). Although these improvements over the Cox model were not statistically significant, they highlight the potential of ensemble learning methods in risk prediction.

**Figure 3:**
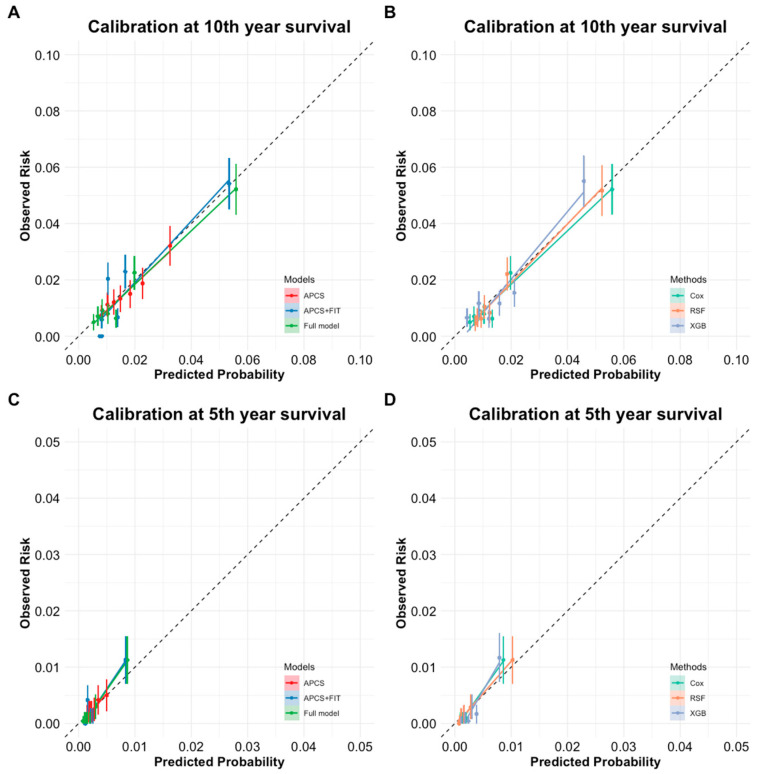
(**A**) Calibration curve for 10-year survival across different models (APCS, APCS + FIT, and Full model) for predicted probability vs. observed risk. (**B**) Calibration curve for 10-year survival across three modeling methods (Cox, RSF, XGBoost) using the full model for predicted probability vs. observed risk. (**C**) Calibration curve for 5-year survival across different models (APCS, APCS + FIT, and full model) for predicted probability vs. observed risk. (**D**) Calibration curve for 5-year survival across three modeling methods (Cox, RSF, XGBoost) using the full model for predicted probability vs. observed risk.

A slight decline in AUC was observed over time (approximately 2% on average), indicating modest temporal attenuation in model performance. All models showed good calibration, with only minor differences. Notably, the RSF model exhibited the most stable calibration slopes across both time horizons, particularly in higher-risk groups (Table 3 and Figure 3).

### 3.4. Population Stratification

**Figure 4:**
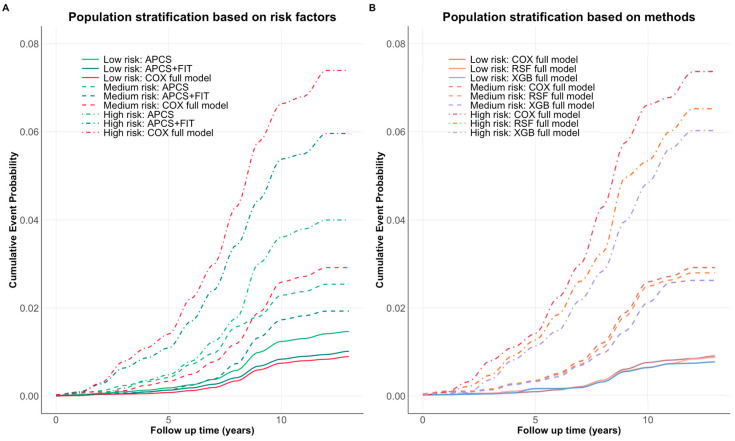
(**A**) Cumulative event probabilities across three models (APCS, APCS + FIT, full model). (**B**) Cumulative event probabilities across three modeling methods (Cox, RSF, XGBoost) using the full model risk factors. Each color represents a specific model, while risk level is distinguished by the line type. All methods demonstrated consistent risk separation over time, with the largest divergence observed in the high-risk group.

The full model demonstrated more distinct separation between risk groups, particularly for high-risk individuals, indicating improved stratification with the inclusion of additional risk factors (Figure 4A). All three methods achieved consistent risk group separation, with only slight variation in predicted cumulative event probabilities (Figure 4B). Supplementary Table S5 reports the sensitivity, specificity, and detection rate of the Cox full model for identifying individuals in the highest-risk quartile. Notably, individuals in the top 20% of predicted absolute risk accounted for 56% of observed CRC cases, with a specificity of 80% and a detection rate of 0.70%. Across models, predicted absolute risks within the same risk quantile were broadly consistent, suggesting robustness in stratification performance.

### 3.5. Decision Curve Analysis

**Figure 5:**
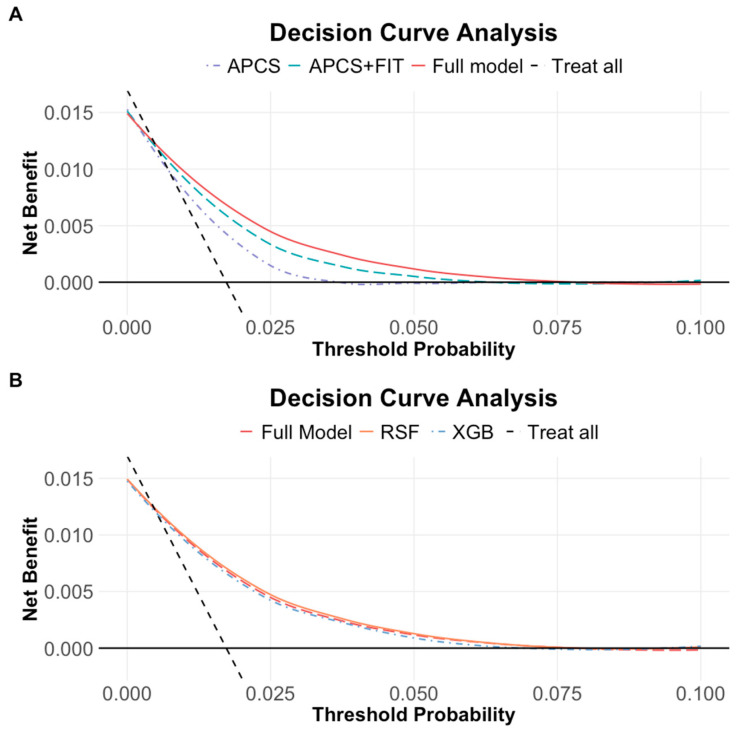
(**A**) Decision curve analysis across three models (APCS, APCS + FIT, full model). (**B**) Decision curve analysis across three modeling methods (Cox, RSF, XGBoost). The solid black line represents a no-screening strategy, and the dashed line represents screening all. At low threshold probabilities, large portions of individuals are recommended for screening, resulting in the highest net benefit. All three models showed comparable net benefit, with performance declining gradually as the threshold increased.

Across a wide range of threshold probabilities, the full model consistently yielded greater net benefit than both the APCS and APCS + FIT models, indicating improved decision-making potential for identifying individuals who would benefit from further diagnostic evaluation. (Figure 5A) While the RSF and XGBoost models showed performance comparable to the Cox-based full model, the differences in net benefit were marginal. (Figure 5B) To illustrate, at a threshold probability of 1%, the net benefit of the Cox full model was 0.008 true positives, corresponding to the detection of 0.8 colorectal cancer cases per 100 individuals, compared to 0.6 with the FIT-enhanced model and fewer with APCS alone. These suggest that the full model could meaningfully improve early cancer detection without substantially increasing unnecessary interventions.

### 3.6. Individual Risk Estimation and Risk Score Calculation

To support individual risk stratification, we derived a linear predictor (risk score) based on the full multivariable Cox model. This score reflects the relative hazard for each participant and enables ranking individuals according to their predicted long-term risk of CRC. The risk score was calculated as a linear combination of the 12 predictors retained in the final model:

$$RiskScore=\sum_{i=1}^{12}\beta_{i}\cdot X_{i}$$

The full set of regression coefficients and variable definitions is provided in Table 2. These estimates may be used to compute individual-level risk scores for population-based stratification and personalized screening strategies. An example formula using the estimated β values is as follows:

RiskScore=1.048×Age+0.548×Female+1.317×Smoker+…+4.872×FITpositive

To estimate the absolute 10-year risk of CRC for each individual, we applied the following formula based on the Cox model framework:

$$\hat{R}\left(10|X\right)=1-\hat{S_{0}}{\left(10\right)}^{\exp\left(RiskScore\right)}$$

where $\hat{S_{0}}\left(10\right)$ denotes the baseline survival probability at 10 years, and the risk score is calculated as described above. In our study, the estimated value of $\hat{S_{0}}\left(10\right)$ was 0.986.

## 4. Discussion

Using data from a large, population-based cohort with over 10 years of follow-up, we developed and validated a 10-year CRC risk prediction model incorporating lifestyle and related factors. The full model showed the best performance across all metrics and offered improved stratification compared to the other three models. It also demonstrates strong applicability to population screening. This stratified approach may enable more precise identification of high-risk individuals, reduce unnecessary screening, and support more efficient allocation of limited healthcare resources. Moreover, integrating dietary and behavioral factors may raise risk awareness and motivate sustained lifestyle changes, contributing to long-term improvements in population health [51,52].

In the risk group analysis, the inclusion of lifestyle risk group and FIT significantly enhanced the discrimination performance of the base model. Notably, although the hazard ratios for the lifestyle related variables showed a relatively narrow range in the Cox regression model, their joint effect contributed substantially to the model’s overall performance. FIT, as a simple and non-invasive CRC screening method itself, also greatly improved the model’s predictive ability, a finding that has been confirmed in several studies. These results suggest the value of incorporating FIT and lifestyle factors in the development of CRC risk prediction models. Information on these modifiable lifestyle factors can facilitate improving health professionals’ ability to advocate for preventive measures and provide preventive health recommendations. Moreover, this tailored approach enables personalized feedback regarding individual lifestyle patterns, which can essentially aid behavior changes in high-risk populations.

When comparing the performance of the three methods in the full model, machine learning approaches exceeded the Cox full model in terms of discrimination ability. However, the extent of the improvement in performance was not statistically significant and should be interpreted with caution. Additionally, we found that the Cox regression model demonstrated predictive capabilities comparable to those of machine learning models in risk stratification and net benefit analysis. This might be attributed to the relatively simple data structure in our study. The full model included only 12 variables and did not exhibit highly complex nonlinear relationships with the outcome. The strength of machine learning approaches typically lies in their ability to capture complex nonlinear relationships and interactions between variables. In a simpler dataset like ours, the traditional Cox proportional hazards model may offer a reliable and more straightforward method, particularly for clinical researchers. Nonetheless, machine learning methods remain valuable for their flexibility and scalability, especially as future datasets incorporate more variables, larger sample sizes, and increasingly complex data structures. It is important to recognize the potential performance improvements provided by machine learning methods.

Our study has several limitations. Firstly, the model was developed using data from the Shanghai program. Risk patterns for colorectal cancer may differ across populations from different regions, ethnicities, and cultural backgrounds. Therefore, future research should validate the model using multi-center datasets to further assess its generalizability and accuracy. Secondly, though we included lifestyle and dietary factors, other important potential risk factors, such as biomarkers and gut microbiota, were not accounted. This limitation primarily stems from the limited accessibility of genetic data. Given that the program began decades ago, obtaining biological samples from large populations was challenging at that time, especially due to high cost and privacy concerns regarding genetic information. However, with advancements in testing methods, recent studies have started exploring large cohorts incorporating genetic factors [53]. However, in the context of large-scale screening, these benefits must be weighed against logistical considerations, cost, and potential ethical concerns. In future studies, further detailed cost-effectiveness analyses and utility assessments based on real-world data are necessary and should be included.

Integrating risk stratification models into existing colorectal cancer screening programs has the potential to improve screening performance and optimize resource allocation. By focusing on high-risk individuals, public health authorities can ensure more efficient use of resources and timely intervention. However, before implementing risk-based screening, it is crucial to conduct a thorough real-world cost-effectiveness evaluation, to assess its clinical benefits and address potential health disparities. Combining lifestyle factors, biomarkers, and genetic information with current screening strategies could further enhance the precision and effectiveness of CRC prevention programs.

## 5. Conclusions

Colorectal cancer is one of the most preventable malignancies and could benefit substantially from risk-stratified screening strategies. We developed and externally validated a 10-year prediction model that demonstrated reasonable discrimination and calibration. By integrating modifiable lifestyle factors, the model enables effective identification of high-risk individuals and provides a practical tool for personalized prevention. Beyond informing targeted screening, it may also enhance individual risk perception and promote positive health behaviors. With further validation and implementation in real-world settings, this model holds promise for integration into population-level screening programs to improve early detection and optimize resource utilization.

## Figures and Tables

**Table 1:** Baseline characteristics of participants in the derivation and validation cohorts.

Characteristics	Overall	Derivation Cohort	Validation Cohort	*p*
*N*	21,358	16,782	4576	
Cases (%)	285 (1.33)	226 (1.35)	59 (1.29)	0.833
Follow up time (median [Q1,Q3])	11.12 (10.09, 11.86)	11.41 (10.55, 12.18)	10.38 [9.86, 11.08]	<0.001
Age (mean (SD))	60.74 (6.99)	60.87 (6.54)	60.24 (8.41)	<0.001
Education (%)				<0.001
Primary (elementary)	4935 (23.1)	4442 (26.5)	493 (10.8)	
Medium (junior and senior)	8004 (37.5)	6216 (37.0)	1788 (39.1)	
High (college and university)	8419 (39.4)	6124 (36.5)	2295 (50.2)	
Smoke (%)				0.835
No	15,860 (74.3)	12,456 (74.2)	3404 (74.4)	
Yes	5498 (25.7)	4326 (25.8)	1172 (25.6)	
Alcohol (%)				0.047
Occasional drinkers	17,654 (82.7)	13,826 (82.4)	3828 (83.7)	
Current drinkers	3704 (17.3)	2956 (17.6)	748 (16.3)	
Red meat (%)				<0.001
≤50 g per day	13,661 (63.9)	10,538 (62.8)	3123 (68.2)	
>50 g per day	7697 (36.0)	6244 (37.2)	1453 (31.8)	
Deep fried food (%)				<0.001
≤3 meals per week	16,492 (77.2)	13,071 (77.9)	3421 (74.8)	
>3 meals per week	4866 (22.8)	3711 (22.1)	1155 (25.2)	
Pickle (%)				<0.001
≤1 meal per week	12,909 (60.4)	9864 (58.8)	3045 (66.5)	
>1 meal per week	8449 (39.6)	6918 (41.2)	1531 (33.5)	
Vegetables and fruits (%)				<0.001
≥300 g per day	3389 (15.9)	2757 (16.4)	632(13.8)	
<300 g per day	17,969 (84.1)	14,025 (83.6)	3944 (86.2)	
BMI (mean (SD))	24.42 (3.26)	24.48 (3.27)	24.22 (3.23)	<0.001
Waist (mean (SD))	82.59 (9.08)	82.89 (9.10)	81.48 (8.92)	<0.001
Hip (mean (SD))	94.68 (6.91)	94.96 (6.96)	93.66 (6.62)	<0.001
Diabetes (%)				<0.001
No	16,986 (79.5)	13,212 (78.7)	3774 (82.5)	
Yes	4372 (20.5)	3570 (21.3)	802 (17.5)	
Diarrhea (%)				
No	20,705 (96.9)	16,200 (96.5)	4505 (98.4)	<0.001
Yes	653 (3.1)	582 (3.5)	71 (1.6)	
Constipation (%)				
No	20,163 (94.4)	15,839 (94.4)	4324 (94.5)	0.798
Yes	1195 (5.6)	943 (5.6)	252 (5.5)	
Hematochezia (%)				
No	20,981 (98.2)	16,467 (98.1)	4514 (98.6)	0.021
Yes	377 (1.8)	315 (1.9)	62 (1.4)	
Mucous (%)				
No	21,145 (99.0)	16,596 (98.9)	4549 (99.4)	0.002
Yes	213 (1.0)	186 (1.1)	27 (0.6)	
Stool deformity (%)				
No	20,531 (96.1)	16,097 (95.9)	4434 (96.9)	0.003
Yes	827 (3.9)	685 (4.1)	142 (3.1)	
Family history (%)				
No	15,461 (72.4)	12,079 (72.0)	3382 (73.9)	<0.001
Yes	5897 (27.6)	4703 (28.0)	1194 (26.1)	
FIT (%)				
Negative	18,237 (85.4)	14,130 (84.2)	4107 (89.8)	<0.001
Positive	3121 (14.6)	2652 (15.8)	469 (10.2)	

Values are presented as n (%) unless otherwise specified. *p* values represent comparisons between the development and validation cohorts. Continuous variables were compared using t-tests or Wilcoxon rank-sum tests as appropriate; categorical variables (including those with multiple categories) were compared using the chi-square test.

**Table 2:** Hazard ratios and confidence intervals for variables included in the full Cox model.

Characteristics	HR	95% CI	*p* Value
Age	1.048	1.029–1.067	<0.001
Gender			
Male	Ref	Ref	
Female	0.548	0.400–0.749	<0.001
Smoke			
No	Ref	Ref	
Yes	1.317	1.011–1.717	0.042
Alcohol			
Occasional drinkers	Ref	Ref	
Current drinkers	1.192	0.799–1.787	0.089
Red meat (%)			
≤2 meals per week	Ref	Ref	
>2 meals per week	1.021	1.003–1.039	0.048
Deep fried food (%)			
≤2 meals per week	Ref	Ref	
>2 meals per week	1.209	0.879–1.663	0.062
Pickle (%)			
≤2 meals per week	Ref	Ref	
>2 meals per week	1.019	0.844–1.232	0.094
Vegetables and fruits (%)			
≥300 g per week	Ref	Ref	
<300 g per week	1.184	0.988–1.148	0.052
Body shape	1.142	1.058–1.388	0.004
Diabetes (%)			
No	Ref	Ref	
Yes	1.317	1.011–1.717	0.043
Family history (%)			
No	Ref	Ref	
Yes	1.164	0.914–1.483	0.218
FIT results (%)			
Negative	Ref	Ref	
Positive	4.877	3.860–6.162	<0.001

**Table 3:** Model performances in training and validation sets.

Model	C-Index				
	Apparent	Bootstrap	Optimism	Optimism-Corrected	Validation
**APCS**	0.634	0.626 (0.589, 0.657)	0.008	0.626	0.622
**FIT + APCS**	0.709	0.699 (0.651, 0.709)	0.010	0.699	0.687
**Cox full model**	0.739	0.727 (0.682, 0.762)	0.012	0.727	0.718
**RSF full model**	0.754	0.734 (0.691, 0.776)	0.020	0.734	0.728
**XGB full model**	0.760	0.739 (0.692, 0.783)	0.021	0.739	0.729
**Model**	**IBS**				
	**Apparent**	**Bootstrap**	**Optimism**	**Optimism-Corrected**	**Validation**
**APCS**	0.008	0.011 (0.007, 0.014)	−0.004	0.012	0.014
**FIT + APCS**	0.008	0.011 (0.008, 0.014)	−0.004	0.012	0.014
**Cox full model**	0.008	0.011 (0.007, 0.016)	−0.004	0.012	0.014
**RSF full model**	0.008	0.010 (0.007, 0.014)	−0.003	0.011	0.014
**XGB full model**	0.008	0.013 (0.008, 0.016)	−0.005	0.013	0.014

Apparent” refers to the model’s performance evaluated directly on the training dataset (*n* = 16,872). “Bootstrap” reflects the mean performance across 1000 bootstrap resamples from the training cohort. Values in parentheses indicate 95% confidence intervals derived from bootstrap resampling. “Optimism” is the difference between apparent and bootstrap performance, representing model overfitting. “Optimism-corrected” indicates internal validation results after adjusting for optimism. “Validation” represents external validation performance assessed in the independent validation cohort (*n* = 4576).

## Data Availability

The data presented in this study are available upon request from the corresponding author. The data is not publicly available due to ethical restrictions and participant confidentiality protections mandated by the informed consent process.

## Supplementary Materials

The following supporting information can be downloaded at https://www.mdpi.com/article/10.3390/nu17111898/s1. Table S1: Missingness Proportions Across All Variables in the Full Dataset; Figure S1: Density Distribution of Observed and Imputed Values for Variables with <30% Missingness; Table S2: The Hyperparameter Search Space of RSF and XGBoost Models; Table S3: Tests of Proportional Hazards Assumption for Covariates in the Full Cox Model; Table S4: Elastic Net Variable Importance Coefficients; Figure S2. Random Survival Forest Variable Importance Scores; Table S5: Sensitivity, Specificity and Detection Rate of Full Model Cox Risk Score for Colorectal Cancer Diagnosis Across the Top 25% of Absolute Risk.
