# Supplementary material for: Development and Validation of a Lifestyle-Based 10-Year Risk Prediction Model of Colorectal Cancer for Early Stratification: Evidence from a Longitudinal Screening Cohort in China
Source: Nutrients. 2025 May 31;17(11):1898. doi: 10.3390/nu17111898 (PMC12158116; doi:10.3390/nu17111898)
Supplement: Supplementary file 1 [file nutrients-17-01898-s001.zip › nutrients-3655733-supplementary.pdf]

Supplementary Materials

Table S1. Missingness Proportions Across All Variables in the Full Dataset

Figure S1. Density Distribution of Observed and Imputed Values for Variables with <30% Missingness

Table S2. The Hyperparameter Search Space of RSF and XGBoost Models

Table S3. Tests of Proportional Hazards Assumption for Covariates in the Full Cox Model

Table S4. Elastic Net Variable Importance Coefficients

Figure S2. Random Survival Forest Variable Importance Scores

Table S5. Sensitivity, Specificity and Detection Rate of Full Model Cox Risk Score for Colorectal Cancer Diagnosis Across the Top 25% of Absolute Risk

Table S1. Missingness Proportions Across All Variables in the Full Dataset

Characteristics	Count (%)	Imputed methods
Education	26 (0.12)	Proportional odds models
Smoke	29 (0.14)	Random forest
Red meat	188 (0.88)	Random forest
Deep fried food	263 (1.23)	Random forest
pickle	191 (0.89)	Random forest

Table S1 shows the percentage of missing values for each variable prior to exclusion and imputation, based on the full cohort of 21,358 participants. Variables with more than 30% missingness were excluded from subsequent analysis.

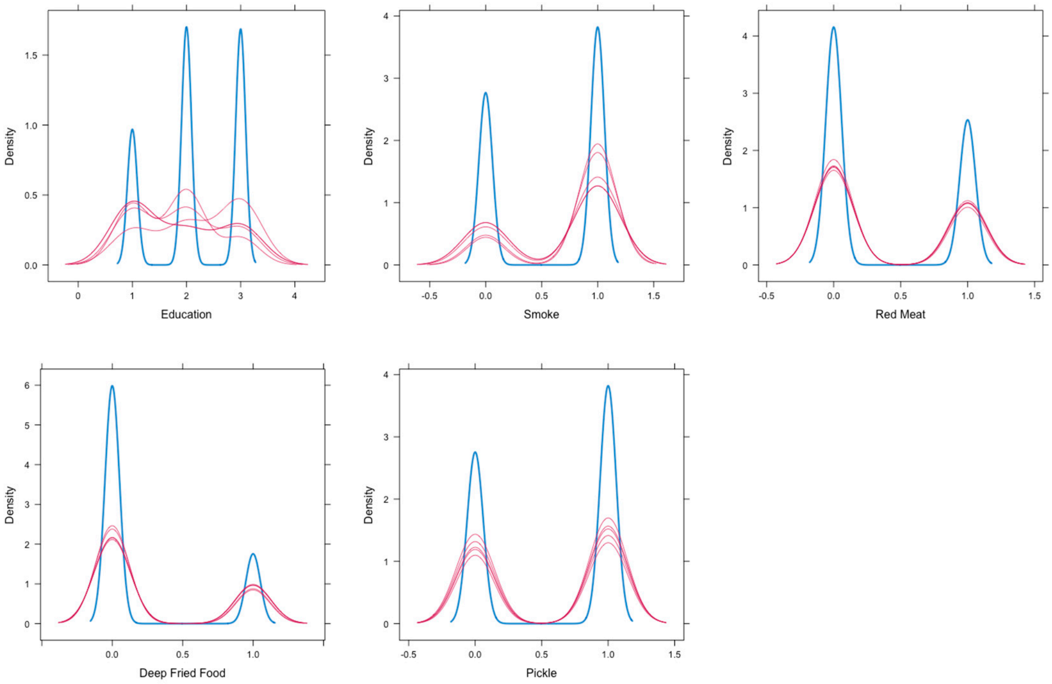

Figure S1 Density Distribution of Observed and Imputed Values for Variables with <30% Missingness. Blue lines represent the density distribution of the observed data, and red lines indicate the distributions from five multiply imputed datasets. The close alignment between the distributions demonstrates the plausibility and consistency of the imputed values.

Table S2. The Hyperparameter Search Space of RSF and XGBoost Models

Model hyper-parameter	Hyper-parameter search space
**RSF**	
n_trees	500, 1000, 2000
n_depth	from 2 to 15, grid = 1
mtry	from 2 to 14, grid = 1
nsplit	from 5 to 30, grid = 1
**XGBoost**	
nrounds	from 100 to 1000, grid = 100
aft_loss_distribution_scale	from 0.5 to 2, grid = 0.05
eta	from 0.001 to 0.05, grid = 0.001
gamma	from 0.1 to 1.5, grid = 0.1
min_child_weight	from 50 to 400, grid = 25
max_depth	from 2 to 15, grid = 1
colsample_bytree	from 0.5 to 1, grid = 0.05
subsample	from 0.6 to 1, grid = 0.05

Table S3 Tests of Proportional Hazards Assumption for Covariates in the Full Cox Model

Characteristics	Chi-square	Degrees of freedom	P value
Age	9.785	1	0.018
Gender	0.469	1	0.493
Smoke	0.196	1	0.658
Alcohol	3.303	1	0.069
Red meat	0.063	1	0.802
Deep fried food	1.074	1	0.300
Pickle	0.055	1	0.814
Vegetables and fruits	0.971	1	0.324
Body shape-Component1	0.447	1	0.504
Diabetes	0.463	1	0.496
Family history	0.533	1	0.465
FIT results	8.068	1	0.045
Global	34.714	14	0.036

Schoenfeld residuals tests were used to assess the proportional hazards (PH) assumption for each covariate and globally for the full Cox model. Among the 12 predictors, age ($p = 0.018$) and FIT result ($p = 0.045$) showed statistically significant deviation from the PH assumption at the 0.05 level. The global test was also significant ($p = 0.036$), suggesting that the overall model may partially violate the PH assumption. However, these variables were retained in the model due to their strong clinical relevance and predictive value. To address potential non-proportionality, machine learning–based survival models were also applied as complementary analyses.

Table S4 Elastic Net Variable Importance Coefficients

Characteristics	Coefficients	Characteristics	Coefficients
Age	0.8152	Body shape-C1	0.6940
Gender	-0.3493	Diabetes	0.5162
Education	.	Diarrhea	.
Smoke	0.7178	Constipation	0.0072
Alcohol	0.6093	Hematochezia	.
Red meat	0.3234	Mucous	.
Deep fried food	0.2017	Stool deformity	0.0400
Vegetables and fruits	-0.2931	FIT	1.3201
Pickles	0.1828	Family history	0.1956

The elastic net variable selection identified FIT as the most influential predictor, with the largest coefficient. While the coefficients for education level, diarrhea, constipation, mucous, and stool deformity were shrunk to zero, indicating that these variables contributed minimally to the model. The other variables also contributed to the model, with gender and fruit intake exhibiting negative associations with the outcome. The optimal parameters for the model were determined through 10-fold cross-validation, with alpha = 0.4 (L1 regularization weight) and lambda = 0.08 (regularization strength).

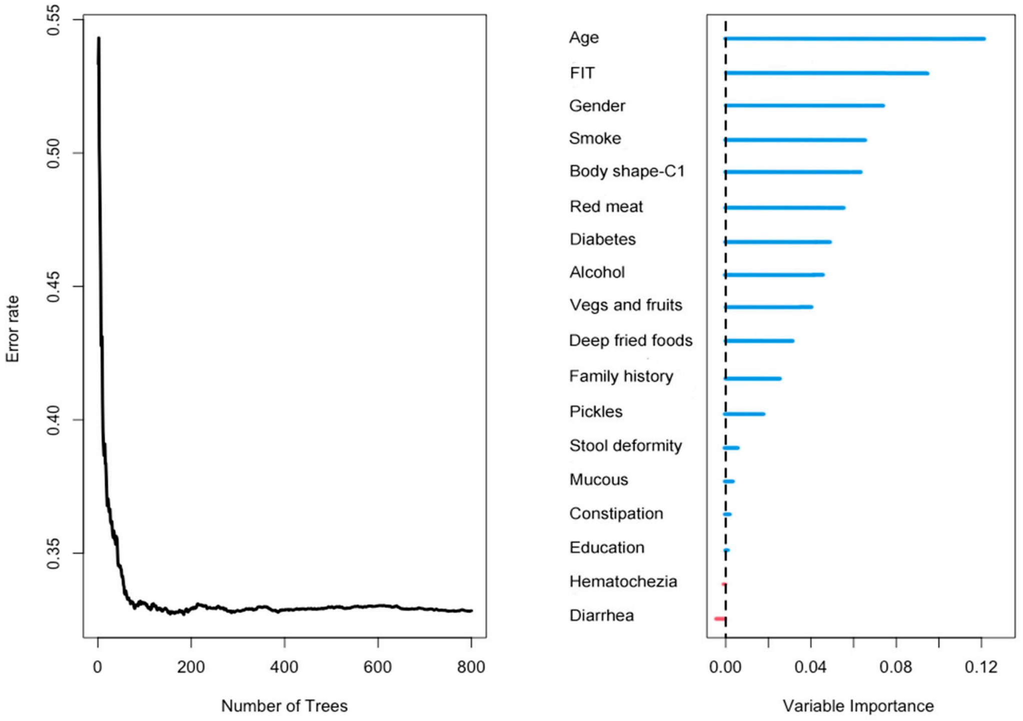

Figure S2 Random Survival Forest Variable Importance Scores. Age, FIT, and gender are the strongest contributors to CRC incidence. Lifestyle factors such as smoking, BMI, alcohol intake, and fruit and vegetable consumption also have notable impacts. Among disease-related factors, diabetes is the most significant, while symptoms like diarrhea and hematochezia contribute little to risk prediction and may even have a negative effect, possibly due to the imbalanced data structure.

Table S5 Sensitivity, Specificity and Detection Rate of Full Model Cox Risk Score for Colorectal Cancer Diagnosis Across the Top 25% of Absolute Risk

Percentage	Population percentage	Absolute 10-year risk centile cut-off	Case per percentage	Sensitivity	Specificity	Detection rate
top 5%	229	6.90	13	0.22	0.95	0.0028
top 10%	228	4.50	8	0.36	0.89	0.0046
top 15%	230	3.09	6	0.46	0.85	0.0059
top 20%	229	2.42	5	0.54	0.80	0.0070
top 25%	233	2.05	2	0.58	0.75	0.0078
